# Supplementary material for: Determination of Urinary Pterins by Capillary Electrophoresis Coupled with LED-Induced Fluorescence Detector
Source: Molecules. 2019 Mar 24;24(6):1166. doi: 10.3390/molecules24061166 (PMC6470587; doi:10.3390/molecules24061166)
Supplement: Supplementary file 1 [file molecules-24-01166-s001.pdf]

Determination of Urinary Pterins by Capillary Electrophoresis Coupled with LED-Induced Fluorescence Detector

Wojciech Grochocki, Magdalena Buszewska-Forajta, Szymon Macioszek and Michał J. Markuszewski *

Department of Biopharmacy and Pharmacodynamic, Medical University of Gdansk, Gdansk, 80-416, Poland; grochoo@gumed.edu.pl (W.G.); magdalena.buszewska-forajta@gumed.edu.pl (M.B.-F.); szymon.macioszek@gumed.edu.pl (S.M.)

* Correspondence: markusz@gumed.edu.pl

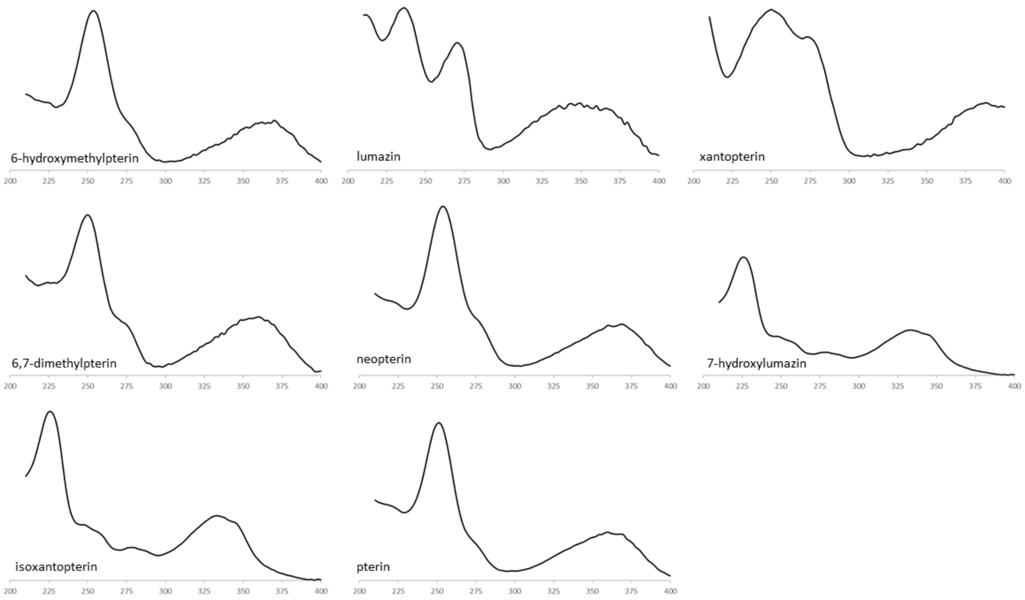

Figure S1. UV absorbance spectra of the selected analytes.
